# Total Cerebral Small Vessel Disease Burden on MRI Correlates With Medial Temporal Lobe Atrophy and Cognitive Performance in Patients of a Memory Clinic

**DOI:** 10.3389/fnagi.2021.698035

**Published:** 2021-09-08

**Authors:** Yangyi Fan, Ming Shen, Yang Huo, Xuguang Gao, Chun Li, Ruimao Zheng, Jun Zhang

**Affiliations:** ^1^Department of Neurology, Peking University People's Hospital, Beijing, China; ^2^Department of Rheumatology and Immunology, Peking University People's Hospital, Beijing, China; ^3^Neuroscience Research Institute, Peking University, Beijing, China

**Keywords:** cerebral small vessel disease, brain magnetic resonance imaging, medial temporal atrophy, cognitive impairment, lacunar, white matter hyperintensities, cerebral microbleed, enlarged perivascular space

## Abstract

**Background:** Cerebral small vessel disease (cSVD) and neurodegeneration are the two main causes of dementia and are considered distinct pathological processes, while studies have shown overlaps and interactions between the two pathological pathways. Medial temporal atrophy (MTA) is considered a classic marker of neurodegeneration. We aimed to investigate the relationship of total cSVD burden and MTA on MRI using a total cSVD score and to explore the impact of the two MRI features on cognition.

**Methods:** Patients in a memory clinic were enrolled, who underwent brain MRI scan and cognitive evaluation within 7 days after the first visit. MTA and total cSVD score were rated using validated visual scales. Cognitive function was assessed by using Mini-Mental State Examination (MMSE) and Montreal Cognitive Assessment (MoCA) scales. Spearman's correlation and regression models were used to test (i) the association between MTA and total cSVD score as well as each cSVD marker and (ii) the correlation of the MRI features and cognitive status.

**Results:** A total of 312 patients were finally enrolled, with a median age of 75.0 (66.0–80.0) years and 40.7% (127/312) males. All of them finished MRI and MMSE, and 293 subjects finished MoCA. Of note, 71.8% (224/312) of the patients had at least one of the cSVD markers, and 48.7% (152/312) of them had moderate–severe MTA. The total cSVD score was independently associated with MTA levels, after adjusting for age, gender, years of education, and other vascular risk factors (OR 1.191, 95% CI 1.071–1.324, *P* = 0.001). In regard to individual markers, a significant association existed only between white matter hyperintensities and MTA after adjusting for the factors mentioned above (OR 1.338, 95% CI 1.050–1.704, *P* = 0.018). Both MTA and total cSVD score were independent risk factors for MMSE ≤ 26 (MTA: OR 1.877, 95% CI 1.407–2.503, *P* < 0.001; total cSVD score: OR 1.474, 95% CI 1.132–1.921, *P* = 0.004), and MoCA < 26 (MTA: OR 1.629, 95% CI 1.112–2.388, *P* = 0.012; total cSVD score: OR 1.520, 95% CI 1.068–2.162, *P* = 0.020). Among all the cSVD markers, microbleed was found significantly associated with MMSE ≤ 26, while no marker was demonstrated a relationship with MoCA < 26.

**Conclusion:** Cerebral small vessel disease was related to MTA in patients of a memory clinic, and both the MRI features had a significant association with cognitive impairment.

## Introduction

Alzheimer's disease (AD) and vascular dementia (VaD) are the most common types of dementia (Plassman et al., 2007). Traditionally, they are considered to have distinct pathological and radiological features. Neurodegeneration is regarded as the main pathology of AD, while vascular disease is the main contributor to VaD. However, growing studies have revealed that there are some overlaps and interactions between the two main pathology pathways. Similar risk factors, such as hypertension, diabetes, hyperlipidemia (Kivipelto et al., 2001; Casserly and Topol, 2004; Vuorinen et al., 2015), and APOEε4 allele gene (Schuur et al., 2011; Sudre et al., 2017; Rojas et al., 2018), have been found in both AD and VaD, which shared common pathophysiological mechanisms including oxidative stress, mitochondrial disruption, and inflammation (Liu et al., 2018). Moreover, previous neuropathological examinations revealed that between 6 and 47% of individuals with dementia had AD and vascular pathologies coexisted (Liu et al., 2015). Recently, molecular imaging *in vivo* showed classic AD biomarkers (amyloid-β and tau) in patients with vascular cognitive impairment (Lee et al., 2011; Yang et al., 2015; Jang et al., 2020).

Cerebral small vessel disease (cSVD) is a progressive syndrome involving the perforating arterioles, capillaries, and venules of the brain. It is thought to be a major cause of VaD (Pantoni, 2010). Till present, the MRI markers for cSVD encompass recent small subcortical infarcts, lacunar infarcts, white matter hyperintensities (WMH), cerebral microbleeds, and enlarged perivascular space (EPVS) (Wardlaw et al., 2013a,b). In contrast, medial temporal atrophy (MTA) is the most widely used MRI marker for neurodegeneration and is related to the diagnosis of AD (Scheltens et al., 1992; Frisoni et al., 2010; Scheltens and van de Pol, 2012). The association of cSVD and MTA as well as their effects on cognitive function have been investigated in several studies, but most of them were focused on individual cSVD markers (mostly WMH), and the results were controversial (van der Flier et al., 2005; Arba et al., 2017; Wang et al., 2021). Recently, a total cSVD score including all the markers mentioned above has been used to assess the total burden of cSVD on MRI and the effects of cSVD on cognitive status (Klarenbeek et al., 2013; Staals et al., 2014, 2015). In this study, we used the total cSVD score on MRI to evaluate the relationship between the total cSVD burden and MTA and explored the impact of these neuroimaging features on the cognitive status of patients in our memory clinic.

## Materials and Methods

### Subjects

Initially, 410 patients with cognitive impairment were retrospectively recruited in the memory clinic of Peking University People's Hospital from January 1, 2018 to December 31, 2020. A total of 337 patients finished MRI scan and got the valid MRI imaging within 7 days after the first visit to the clinic. The following patients were excluded: (1) patients with cognitive dysfunction secondary to central nervous infection, brain trauma, brain tumor, and brain injury caused by radiotherapy or chemotherapy; (2) patients with cognitive impairment caused by metabolic, nutritional, and infectious factors, such as thyroid dysfunction, vitamin B deficiency, alcoholic brain damage, and syphilis; (3) patients with severe psychiatric disease or severe psychiatric symptoms which disturbed the cognitive assessment; (4) patients with large vessel infarctions or severe hemorrhagic stroke (due to the difficulty in MRI assessment for cSVD markers) or patients with WMH on MRI due to leukodystrophy, demyelinating disease of central nervous disease, and vasculitis. Finally, there were 312 patients included in this study. All the patients underwent a clinical evaluation and neurological examination, and the following information was recorded: age, gender, years of education, history of previous stroke, hypertension, diabetes mellitus, hyperlipidemia, coronary heart disease, and smoking. Blood tests about fasting glucose, triglycerides, total cholesterol, low-density lipoprotein cholesterol, high-density lipoprotein cholesterol, and uric acid were also recorded. The clinical initial diagnosis was made at the memory clinic after evaluating the clinical data, cognition function, MRI, and blood tests. Dementia was defined using the Diagnostic and Statistical Manual of Mental Disorders IV (DSM-IV) criteria (American Psychiatric Association, 1994). Probable AD was defined by the National Institute of Neurological and Communicative Disorders and Stroke and the Alzheimer's Disease and Related Disorders Association (NINCDS-ADRDA) criteria (McKhann et al., 1984). Probable VaD was defined by the National Institute of Neurological Disorders and Stroke and the Association Internationale pour la Recherche et l'Enseignement en Neurosciences (NINDS-AIREN) criteria (Román et al., 1993). Mild cognitive impairment (MCI) was defined using the revised MCI criteria in 2003 (Winblad et al., 2004). Subjective cognitive decline (SCD) was defined according to the Subjective Cognitive Decline Initiative (SCD-I) criteria (Molinuevo et al., 2017).

### Cognitive Assessment

The cognitive assessment was performed by a trained neuropsychology using the Chinese version of the Mini-Mental State Examination (MMSE) scale (Folstein et al., 1975) and Montreal Cognitive Assessment (MoCA) scale (Nasreddine et al., 2005) at the first visit. Patients were considered to have cognitive impairment when MMSE ≤ 26 or MoCA < 26 (one additional point was given to patients having <12 years of education for the MoCA scale).

### MRI Imaging

Brain MRI was performed with a 3.0 T scanner (GE 750 or GE 750W, WI, USA) in the radiology department of Peking University People's Hospital within 7 days after their first visit. The sequences of MRI included the T1-weighted, T2-weighted, fluid-attenuated inversion recovery (FLAIR), diffusion-weighted (DWI), and susceptibility-weighted imaging (SWI). The total cSVD score was rated following the STRIVE recommendations (Klarenbeek et al., 2013; Wardlaw et al., 2013b; Staals et al., 2014). Lacunar infarcts were defined as lesions with the cerebrospinal fluid-like signal on all sequences, the diameter of which should be between 3 and 20 mm, with the location in the territory of a perforating arteriole (Wardlaw et al., 2013a). If there were one or more lacunar infarcts, one point was added. WMH was assessed using the Fazekas scale on FLARI (Fazekas et al., 1987), and one point was added when the deep WMH Fazekas score reached 2 or periventricular WMH Fazekas score reached 3. EPVS was defined as cerebrospinal fluid-like lesions with an ovoid, round, or linear shape and a diameter < 3 mm. If the number of EPVS at the basal ganglia level reached 10, one point was added (Doubal et al., 2010). Microbleeds were identified as a homogeneous lesion (diameter < 10 mm) with low signal intensity on SWI, and if there were one or more microbleeds, one point was added (Staals et al., 2009; Doubal et al., 2010). Finally, a total score of cSVD was obtained with a scale from 0 to 4. MTA was rated using a validated visual scale from 0 to 4 on coronal T1 sequence, and patients were classified into the following two groups according to the levels of MTA: none–mild atrophy (levels 0–1) and moderate–severe atrophy (levels 2–4) (Scheltens et al., 1992).

The images were assessed by two neurological radiologists independently who were blind to each other's reading and the clinical information. A consultation was conducted to reach an agreement when there was a divergence.

### Statistical Analysis

The data analysis was performed using the SPSS 19.0 software (IBM Corp., Armonk, NY). When *P* < 0.05, the difference was considered statistically significant. After the normality tests for the data, median [interquartile range (IQR)] was used to present the data of continuous variables, and the Kruskal–Wallis test was chosen to analyze the difference between groups. The χ^2^-test was used for comparing the difference between groups for date of categorical variables [presented as *n* (%)]. The Spearman's correlation analysis was performed to evaluate the relationship between total cSVD score and MTA and the correlation between MRI features (total cSVD score and MTA) and cognitive status (MMSE and MoCA score). We investigated whether the total cSVD score or any of the individual cSVD markers were independent risk factors for MTA using a generalized linear model. Binary logistic regression was used to explore whether total cSVD score and MTA were associated with the cognitive impairment. Classic risk factors for cognitive impairment including gender, age, and education were adjusted, and other vascular risk factors, such as the history of hypertension, diabetes, hyperlipidemia, and cerebrovascular disease, were also adjusted.

## Results

**Figure 1:**
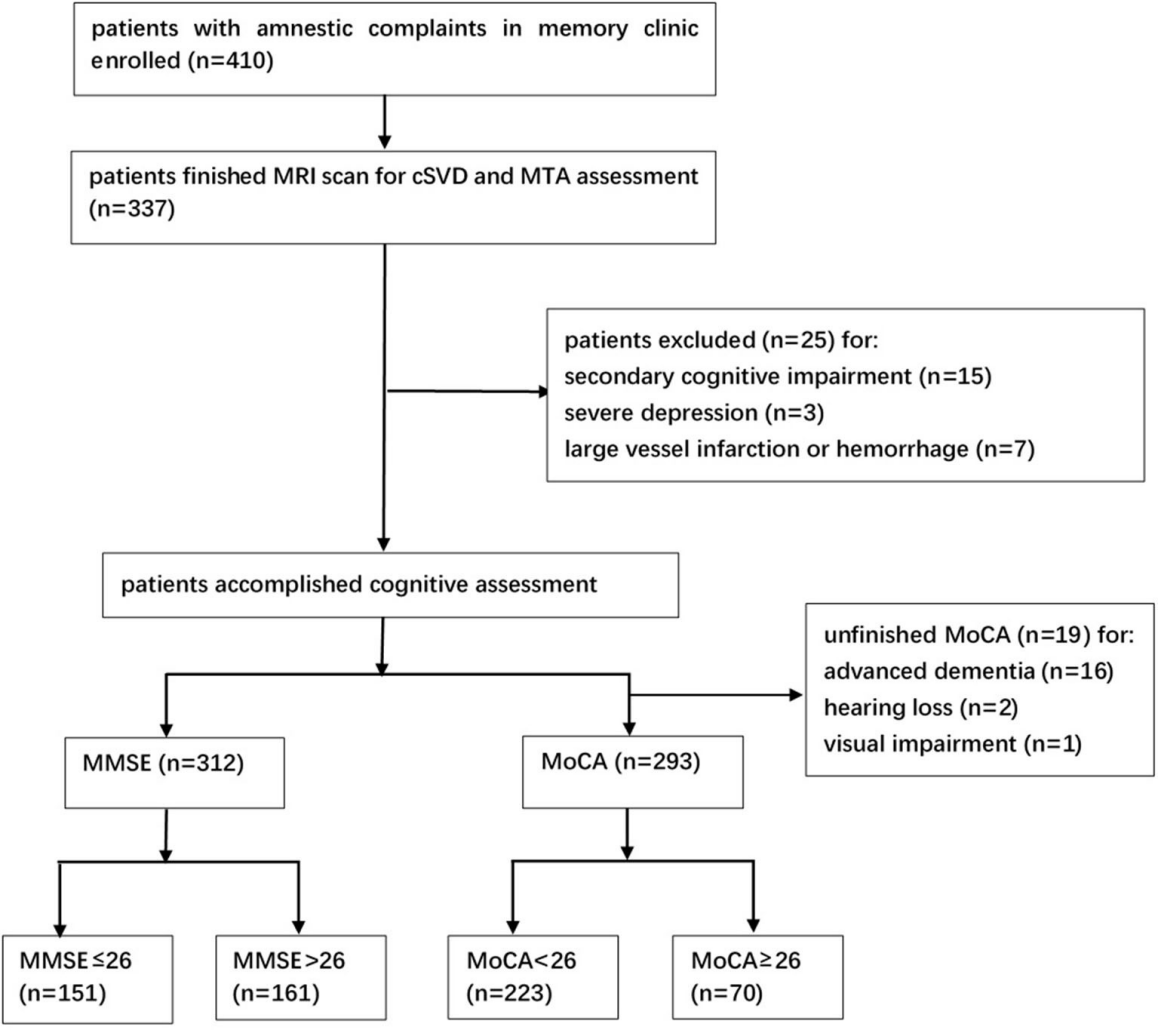
Study flowchart. MRI, magnetic resonance imaging; cSVD, cerebral small vessel disease; MTA, medial temporal atrophy; MMSE, Mini-Mental State Examination; MoCA, Montreal Cognitive Assessment.

A total of 337 patients were enrolled with full clinical, cognitive, and MRI data. Of note, 25 patients were excluded due to brain trauma (2 patients), brain injury due to chemotherapy (2 patients), hypothyroidism (5 patients), vitamin B deficiency (2 patients), alcoholic brain damage (4 patients), severe depression (3 patients), and large vessel infarctions or severe cerebral hemorrhage (7 patients), and finally, 312 patients were recruited (Figure 1). The median age of the participants was 75.0 (66.0–80.0) years, and 40.7% (127/312) of them were males. The median year of education was 15.0 (9.0–15.0), and the median duration before visiting the memory clinic was 1.9 (1.0–3.0) years. Of note, 38.5% (120/312) of the subjects were diagnosed with dementia clinically. 50.8% (61/120) of the patients with dementia were diagnosed with probable AD, 9.2% (11/120) were diagnosed with probable VaD, and 40.0% (48/120) were diagnosed with other types or untyped dementia. Of note, 52.9% (165/312) of patients were diagnosed with MCI, and 8.7% (27/312) of patients were diagnosed with subjective cognitive impairment. Hypertension was noticed in 50.6% (158/312) of the patients, diabetes mellitus in 25.6% (80/312), hyperlipidemia in 13.5% (42/312), coronary heart disease in 14.7% (46/312), and previous stroke in 9.0% (28/312). Of note, 15.1% (47/312) of them were a former or current smoker, and 4.5% (14/312) of them had a family history of dementia. All the patients had the MMSE scale, and the median score was 27.0 (22.0–29.0). A total of 293 patients finished the MoCA scale with a median score of 22.0 (18.0–25.0). The main reason for the uncompleted MoCA was poor cognitive function and coordination. Sixteen of them did not finish MoCA due to advanced dementia [the median MMSE score for them was 10.5 (9.0–13.3)]. Two patients did not finish MoCA due to the very poor hearing (MMSE 18 and 16) and one patient due to the severe visual impairment caused by cataract (MMSE 22).

### Relationship Between cSVD and MTA

On MRI, lacunar infarction was found in 53.8% (168/312) of the patients, >10 EPVS in 37.8% (118/312), deep microbleed in 14.4% (45/312), and moderate-to-severe WMH in 32.3% (101/312). The total cSVD score was 0 point in 28.2% (88/312), 1 point in 27.9% (87/312), 2 points in 24.4% (76/312), 3 points in 16.3% (51/312), and 4 points in 3.2% (10/312) of the participants. Of note, 51.3% (160/312) of the individuals had none–mild MTA (levels 0–1), and 48.7% (152/312) of them had moderate–severe MTA (levels 2–4). Compared to the patients with none–mild MTA, the ones with moderate–severe MTA were older [78.0 (73.0–82.0) vs. 68.0 (62.0–76.8), *P* < 0.001], had a higher total cSVD score [2.0 (1.0–3.0) vs. 1.0 (0–2.0), *P* < 0.001], and had a higher percentage of all the cSVD markers on MRI (Table 1). There was no significant difference between the two groups on sex ratio, years of education, suffering duration before visiting, and the frequency of other vascular risk factors.

**Table 1:** Demographic, clinical, and cSVD characteristics of patients in different MTA groups.

**Variables**	**MTA 0-1**	**MTA 2-4**	***P***
	**(*N* = 160)**	**(*N* = 152)**	
Age (years)	68.0 (62.0–76.8)	78.0 (73.0–82.0)	**<0.001**
Gender (male), *n* (%)	61 (38.1)	66 (43.4)	0.341
Years of education	12.0 (9.0–15.0)	15.0 (9.0–15.0)	0.600
Duration before visiting (years)	1.5 (1.0–3.0)	2.0 (1.0–3.0)	0.235
Hypertension, *n* (%)	74 (46.3)	84 (55.3)	0.111
Diabetes, *n* (%)	43 (26.9)	37 (24.3)	0.609
Hyperlipidemia, *n* (%)	27 (16.9)	15 (9.9)	0.070
Coronary heart disease, *n* (%)	27 (16.9)	19 (12.5)	0.276
Previous stroke, *n* (%)	15 (9.4)	13 (8.6)	0.799
Smoking, *n* (%)	28 (17.5)	19 (12.5)	0.217
Family history, *n* (%)	6 (3.8)	8 (5.3)	0.519
Total cSVD score	1.0 (0–2.0)	2.0 (1.0–3.0)	**<0.001**
EPVS, *n* (%)	49 (30.6)	69 (45.4)	**0.007**
Lacunar, *n* (%)	66 (41.3)	102 (67.1)	**<0.001**
Microbleed, *n* (%)	13 (8.1)	32 (21.1)	**0.001**
WMH, *n* (%)	36 (22.5)	65 (42.8)	**<0.001**

*cSVD, cerebral small vessel disease; MTA, medial temporal atrophy; EPVS, enlarged perivascular space; WMH, white matter hyperintensities*.

*Bold values: P < 0.05*.

The Spearman's correlation analysis revealed that there was a significant correlation between the total cSVD score and the level of MTA (*r* = 0.370, *P* < 0.001). Moreover, a significant correlation was found between each of the cSVD markers and the level of MTA (Table 2). In generalized linear regression models, the total cSVD score was an independent factor for MTA (OR 1.432, 95% CI 1.295–1.582, *P* < 0.001). The association remained significant after adjusting for age, gender, years of education, and other risk factors including hypertension, diabetes, hyperlipidemia, and previous stroke (OR 1.191, 95% CI 1.071–1.324, *P* = 0.001) (Table 3). When all the single markers of cSVD, instead of the total score, were taken into the regression models, lacunar infarction (OR 1.482, 95% CI 1.161–1.893, *P* = 0.002), microbleed (OR 1.450, 95% CI 1.034–2.033, *P* = 0.031), and WMH (OR 1.611, 95% CI 1.244–2.085, *P* < 0.001) were found to have a significant association with MTA, but after adjusting for the factors mentioned above, a significant association existed only between WMH and MTA (OR 1.338, 95% CI 1.050–1.704, *P* = 0.018) (Table 3).

**Table 2:** Correlation analysis of cSVD and MTA.

**cSVD**	***r***	***P***
Total cSVD score	0.370	**<0.001**
EPVS	0.213	**<0.001**
Lanucar	0.270	**<0.001**
Microbleed	0.186	**0.001**
WMH	0.264	**<0.001**

*cSVD, cerebral small vessel disease; MTA, medial temporal atrophy; EPVS, enlarged perivascular space; WMH, white matter hyperintensities*.

*Bold values: P < 0.05*.

**Table 3:** CSVD in relation to MTA by regression analysis.

	**Model 1**		**Model 2**		**Model 3**	
	**OR (95% CI)**	***P***	**OR (95% CI)**	***P***	**OR (95% CI)**	***P***
Total cSVD score	1.432 (1.295–1.582)	**<0.001**	1.166 (1.050–1.294)	0.004	1.191 (1.071–1.324)	**0.001**
EPVS	1.243 (0.963–1.604)	0.095	0.958 (0.754–1.217)	0.725	0.984 (0.774–1.251)	0.898
Lacunar	1.482 (1.161–1.893)	**0.002**	1.199 (0.956–1.504)	0.117	1.232 (0.982–1.547)	0.072
Microbleed	1.450 (1.034–2.033)	**0.031**	1.302 (0.958–1.770)	0.092	1.312 (0.965–1.784)	0.083
WMH	1.611 (1.244–2.085)	**<0.001**	1.318 (1.037–1.675)	0.024	1.338 (1.050–1.704)	**0.018**

*Model 1 for total cSVD score or each marker of cSVD; Model 2. Model 1 + age, gender, and education; Model 3. Model 2 + hypertension, diabetes mellitus, hyperlipidemia, and previous stroke*.

*cSVD, cerebral small vessel disease; MTA, medial temporal atrophy; EPVS, enlarged perivascular space; WMH, white matter hyperintensities*.

*Bold values: P < 0.05*.

### Impact of cSVD and MTA on Cognitive Status

A total of 161 (51.6%) participants had the MMSE score > 26, while 151 (48.4%) of them had MMSE ≤ 26, suggesting cognitive impairment. Compared with the former ones, those with MMSE ≤ 26 were older [78.0 (68.0–82.0) vs. 72.0 (65.0–78.0), *P* < 0.001], less educated [12.0 (9.0–15.0) vs. 15.0 (12.0–15.0), *P* < 0.001], with a longer duration before visiting [2.0 (1.0–3.0) vs. 1.5 (0.6–2.8), *P* = 0.010], had higher MTA levels [2.0 (1.0–3.0) vs. 1.0 (0–2.0), *P* < 0.001], and cSVD scores [2.0 (1.0–3.0) vs. 1.0 (0–2.0), *P* < 0.001]. Besides, they had all the cSVD markers more frequently than those with MMSE score > 26. No significant difference was found between the two groups on sex ratio and other vascular risk factors (Table 4). Significant correlation was found between MTA level and MMSE score (*r* = 0.424, *P* < 0.001), and between total cSVD score and MMSE score (*r* = 0.356, *P* < 0.001) through the Spearman's correlation analysis. Binary logistic regression demonstrated both MTA (OR 1.877, 95% CI 1.407–2.503, *P* < 0.001) and total cSVD score (OR 1.474, 95% CI 1.132–1.921, *P* = 0.004) were independent risk factors for cognitive impairment (MMSE ≤ 26), even after adjusting for age, gender, years of education, duration before visiting, hypertension, diabetes, hyperlipidemia, and previous stroke (Table 5). When individual markers were included in the regression instead of the total cSVD score, only microbleed was significantly associated with cognitive impairment (MMSE ≤ 26) (OR 3.877, 95% CI 1.646–9.135, *P* = 0.002) after adjustment for the confounding factors mentioned above (Table 5).

**Table 4:** Demographic, clinical, and MRI characteristics of patients in different cognition status groups.

**Variables**	**MMSE > 26**	**MMSE ≤ 26**	***P***	**MoCA ≥ 26**	**MoCA < 26**	***P***
	**(*N* = 161)**	**(*N* = 151)**		**(*N* = 70)**	**(*N* = 223)**	
Age (years)	72.0 (65.0–78.0)	78.0 (68.0–82.0)	**<0.001**	66.5 (59.0–75.3)	76.0 (68.0–80.0)	**<0.001**
Gender (male), *n* (%)	69 (42.9)	58 (38.4)	0.424	32 (45.7)	87 (39.0)	0.319
Years of education	15.0 (12.0–15.0)	12.0 (9.0–15.0)	**<0.001**	15.0 (12.0–16.0)	15.0 (9.0–15.0)	**<0.001**
Duration before visiting (years)	1.5 (0.6–2.8)	2.0 (1.0–3.0)	**0.010**	1.0 (0.5–2.0)	2.0 (1.0–3.0)	**0.014**
Hypertension, *n* (%)	77 (47.8)	81 (53.6)	0.304	37 (52.9)	113 (50.7)	0.750
Diabetes, *n* (%)	44 (27.3)	36 (23.8)	0.481	21 (30.0)	53 (23.8)	0.295
Hyperlipidemia, *n* (%)	24 (14.9)	18 (11.9)	0.440	10 (14.3)	29 (13.0)	0.783
Coronary heart disease, *n* (%)	21 (13.0)	25 (16.6)	0.382	9 (12.9)	32 (14.3)	0.753
Previous stroke, *n* (%)	11 (6.8)	17 (11.3)	0.172	1 (1.4)	23 (10.3)	**0.018**
Smoking, *n* (%)	30 (18.6)	17 (11.3)	0.069	13 (18.6)	29 (13.0)	0.246
Family history, *n* (%)	10 (6.2)	4 (2.6)	0.129	5 (7.1)	8 (3.6)	0.208
MTA level	1.0 (0–2.0)	2.0 (1.0–3.0)	**<0.001**	1.0 (0–2.0)	2.0 (1.0–3.0)	**<0.001**
Total cSVD score	1.0 (0–2.0)	2.0 (1.0–3.0)	**<0.001**	0 (0–1.0)	1.0 (1.0–2.0)	**<0.001**
EPVS, *n* (%)	49 (30.4)	69 (45.7)	**0.005**	14 (20)	93 (41.7)	**0.001**
Lacunar, *n* (%)	69 (42.9)	99 (65.6)	**<0.001**	24 (34.3)	130 (58.3)	**<0.001**
Microbleed, *n* (%)	9 (5.6)	36 (23.8)	**<0.001**	1 (1.4)	39 (17.5)	**0.001**
WMH, *n* (%)	37 (23.0)	64 (42.4)	**<0.001**	10 (14.3)	81 (36.3)	**0.001**

*MMSE, Mini-Mental State Examination; MoCA, Montreal Cognitive Assessment; MTA, medial temporal atrophy; cSVD, cerebral small vessel disease; EPVS, enlarged perivascular space; WMH, white matter hyperintensities*.

*Bold values: P < 0.05*.

**Table 5:** MTA and cSVD in relation to cognitive status by regression analysis.

	**Model 1**		**Model 2**		**Model 3**	
	**OR (95% CI)**	***P***	**OR (95% CI)**	***P***	**OR (95% CI)**	***P***
**MMSE ≤ 26**						
MTA	1.713 (1.348–2.176)	**<0.001**	1.847 (1.391–2.452)	**<0.001**	1.877 (1.407–2.503)	**<0.001**
Total cSVD score	1.592 (1.264–2.006)	**<0.001**	1.511 (1.167–1.957)	**0.002**	1.474 (1.132–1.921)	**0.004**
EPVS	1.054 (0.614–1.809)	0.848	1.042 (0.581–1.868)	0.891	1.029 (0.570–1.857)	0.925
Lacunar	1.525 (0.909–2.560)	0.110	1.387 (0.799–2.408)	0.244	1.341 (0.768–2.342)	0.302
Microbleed	3.947 (1.731–9.003)	**0.001**	3.985 (1.705–9.314)	**0.001**	3.877 (1.646–9.135)	**0.002**
WMH	1.754 (1.007–3.055)	**0.047**	1.530 (0.850–2.752)	0.156	1.493 (0.818–2.723)	0.191
**MoCA < 26**						
MTA	1.750 (1.287–2.381)	**<0.001**	1.593 (1.100–2.307)	**0.014**	1.629 (1.112–2.388)	**0.012**
Total cSVD score	1.856 (1.354–2.543)	**<0.001**	1.572 (1.115–2.217)	**0.010**	1.520 (1.068–2.162)	**0.020**
EPVS	1.520 (0.744–3.108)	0.251	1.398 (0.655–2.983)	0.386	1.384 (0.634–3.019)	0.415
Lacunar	1.431 (0.765–2.676)	0.261	1.130 (0.576–2.214)	0.723	1.111 (0.558–2.212)	0.765
Microbleed	9.574(1.244–73.695)	**0.030**	8.434 (1.089–65.293)	**0.041**	7.046 (0.900–55.186)	0.063
WMH	2.156 (0.988–4.707)	0.054	1.761 (0.773**–**4.011)	0.178	1.664 (0.714–3.878)	0.238

*Model 1 for MTA and total cSVD score; Model 2. Model 1+ age, gender, education, and duration before visiting; Model 3. Model 2 + hypertension, diabetes mellitus, hyperlipidemia, and previous stroke*.

*MMSE, Mini-Mental State Examination; MoCA, Montreal Cognitive Assessment; MTA, medial temporal atrophy; cSVD, cerebral small vessel disease; EPVS, enlarged perivascular space; WMH, white matter hyperintensities*.

*Bold values: P < 0.05*.

Similar results were found when the MoCA scale was used, which was supposed to be better for identifying MCI. Patients with MoCA < 26 [76.1% (223/293)] were older [76.0 (68.0–80.0) vs. 66.5 (59.0–75.3), *P* < 0.001], had less education years [15.0 (9.0–15.0) vs. 15.0 (12.0–16.0), *P* < 0.001], longer suffering time before visiting [2.0 (1.0–3.0) vs. 1.0 (0.5–2.0), *P* = 0.014], and higher percentage of previous stroke [10.3% (23/223) vs. 1.4% (1/70), *P* = 0.018] than those with MoCA ≥ 26 [23.9% (70/293)]. They also had a higher MTA level [2.0 (1.0–3.0) vs. 1.0 (0–2.0), *P* < 0.001], total cSVD score [1.0 (1.0–2.0) vs. 0 (0–1.0), *P* < 0.001], and frequency of having all the individual cSVD markers (Table 4). The Spearman's correlation analysis illustrated a significant correlation between MTA level and MoCA score (*r* = 0.424, *P* < 0.001) and between total cSVD score and MoCA score (*r* = 0.378, *P* < 0.001). Both MTA (OR 1.629, 95% CI 1.112–2.388, *P* = 0.012) and total cSVD score (OR 1.520, 95% CI 1.068–2.162, *P* = 0.020) revealed significant independent risk factors for impaired cognitive function (MoCA < 26) in binary logistic regression, after correcting the confounding factors mentioned in regression for MMSE (Table 5). But none of the markers was found significantly associated with MoCA < 26 when they were analyzed separately instead of total cSVD score (Table 5).

## Discussion

In this study, we investigated the relationship between MTA and total cSVD burden on MRI as well as the effects of the two radiological features on the cognitive performance of patients from a memory clinic. We found a significant correlation between MTA levels and total cSVD score, and the total cSVD score was a significant risk factor for MTA. Moreover, both MTA and total cSVD score were independent risk factors for cognitive impairment, no matter assessed by MMSE or MoCA. Although previous studies have explored the relationship of cSVD and MTA together with their impact on cognition, this study is one of the few analyzing such relationship and impaction using total cSVD score to describe cSVD in a relatively comprehensive way.

We recruited a group of patients with amnestic complaints, but with older age and higher education than previous studies (Arba et al., 2017; Banerjee et al., 2018). Most of them showed mild to moderate cognitive impairment. cSVD was found in 71.8% (224/312) of them and moderate–severe MTA in 48.7% (152/312) of them. Referring to the relationship of cSVD and MTA, previous study was mostly focused on WMH, and a significant correlation was observed (van der Flier et al., 2005; Akinyemi et al., 2015; Arba et al., 2017; Wong et al., 2019; Wang et al., 2021), while investigations about other cSVD markers were limited and the results were inconsistent (Wang et al., 2021). The total cSVD score has been tried in several studies about cSVD and cognitive function (Staals et al., 2014; Uiterwijk et al., 2016; Banerjee et al., 2018; Del Brutto et al., 2018). Although the dichotomization in the scoring of each marker would lead to a loss of information, it was a relatively comprehensive method to assess the total cSVD burden (Wardlaw et al., 2013b; Staals et al., 2015). We revealed a significant positive correlation between total cSVD score and MTA, with a higher total cSVD burden among the ones with higher MTA levels. With regard to every individual marker, after adjusting the confounding factors, only WMH was found to be a significant risk factor for MTA. It indicated that WMH might be the main factor in cSVD accounting for neurodegeneration and MTA, which was echoed by plenty of studies (van der Flier et al., 2005; Kloppenborg et al., 2012; Akinyemi et al., 2015; Arba et al., 2017; Wong et al., 2019; Wang et al., 2021). Several interpretations have been proposed to explain the relationship of WMH and MTA: first, the medial temporal lobe is vulnerable to hypoperfusion and ischemia while WMH is usually with reduced global blood flow, and the association between MTA and WMH is the result of the common vascular-ischemic pathology (Schmidt-Kastner and Freund, 1991; de la Torre, 2004; Nishio et al., 2010). Second, the damage of vascular integrity and blood–brain barrier found in WMH leads to neuron damage of the hippocampus (Young et al., 2008). Finally, WMH causes cortical disconnection and damage in tracts serving the medial temporal lobe, which may cause subsequent MTA (Villain et al., 2008; Fiford et al., 2017). Other cSVD features were not identified as significant factors for MTA in our research, which was inconsistent with the results of limited previous studies (Thong et al., 2013; Montandon et al., 2020). That might result from the “all-or-non” pattern for assessing each individual marker in this point-scoring system, which led to the information about the severity of every individual cSVD marker being partially missed. A scoring system comprising all the cSVD features and including more quantitative analyses is supposed in future research.

In this study, both MTA severity and total cSVD score on MRI were independent risk factors for cognitive impairment, no matter evaluating by MMSE or MoCA. It provided further evidence for the perspective that there was an overlap and synergistic effect between neurovascular and neurodegenerative pathology for cognitive impairment (Jokinen et al., 2012). MTA was a proven predictor for cognitive decline in kinds of cohorts including subjects in various stages of cognitive impairment, whether with cerebrovascular disease or not (Mungas et al., 2001, 2005; Cardenas et al., 2011; Kebets et al., 2015). In our research, MTA was also the strongest risk factor for cognitive dysfunction, which was consistent with the past observations. Besides, the total cSVD score was another significant factor for cognitive impairment, even only 9.0% (28/312) of the patients had a history of stroke in our cohort. It suggested that small vessel disease may play an important role in cognitive decline in a more widespread way, which was verified in several studies recently (Uiterwijk et al., 2016; Banerjee et al., 2018; Del Brutto et al., 2018). A cumulative effect of MTA and cSVD burden was found in our study, with the subjects with both MTA and cSVD features having the highest risk for cognitive impairment. The synergistic interactions of MTA and cSVD have been demonstrated in previous studies, but only about separate cSVD features such as WMH and lacunar (van der Flier et al., 2005; Jokinen et al., 2012; Wong et al., 2019). Our results extended the previous observations by evaluating the total cSVD burden in a more overall way. When separate markers were analyzed in the regression, only microbleed showed significant association with MMSE ≤ 26. Microbleed has demonstrated a risk factor for cognitive decline in recent studies (Akoudad et al., 2016; Montandon et al., 2020), and our results in the MMSE score provided further support for the point of view. The inconsistency on the MoCA scale might be due to the different sensitivity for mild cognitive impairment of the two scales (Nasreddine et al., 2005). In contrast, in our opinion, the insignificance in the regression about the other cSVD markers did not mean that there was no impact of them on cognitive dysfunction. The information loss about the severity mentioned above was a major reason, and there might be an accumulative effect for all the markers in cognitive impairing which led to the final significant impact of the total cSVD burden.

The strengths of this study were as follows: we used a more comprehensive method to evaluate cSVD burden and MTA in standardized scales, with good feasibility and reproducibility in clinical practice. It concentrated on the effect of the total cSVD burden on MTA and cognition instead of separate cSVD markers. We chose the widely accepted MMSE and MoCA scales and focused on the overall cognitive function rather than the definite diagnosis. This was more fitted with daily clinical practice and real-world research because some examinations for definite diagnosis (e.g., cerebrospinal fluid Aβ42, tau testing, and molecular imaging) were not easily available in the real world. The results indicated the necessity and feasibility to include both of the two features in the radiological assessment for patients with cognitive impairment in clinical practice. Moreover, the results also offered evidence for the need to incorporate treatments for neurodegeneration and reduction of the cSVD burden in future work. In contrast, there were realistic limitations of this study in several aspects. It was a retrospective, cross-sectional study that enrolled a certain population in a single center. This limited the extension of our conclusion to the broader population, and the lack of follow-up affected the assessment for the evolution of cognitive impairment longitudinally. More multicenter, longitudinal studies with a large sample size are needed in the future to understand the relationship and the roles of MTA and cSVD burden in cognitive deterioration. The visual rating for the radiological assessment could lead to some extent of subjectivity, and as mentioned above, the “all-or-non” pattern in scoring each individual marker of cSVD induced some information missed. Besides, more specific features for neurodegeneration such as Aβ imaging and more quantitative measurement for cSVD burden (such as the volume of WMH) should be included in future research to explore the pathology of neurodegeneration and vascular disease. Although the focus on overall function using screening scales was preferable in daily clinical work, studies about subjects selected with a more specific diagnosis are needed to remove the interference factors, and more specific neuropsychological tests should be included to explore the impairment of different cognitive domains in patients with different MTA and cSVD levels.

## Conclusion

This study provided further evidence about the correlation of cSVD and MTA in patients with cognitive dysfunction. It supported the perspective that there was interaction and combination in vascular and neurodegenerative pathology in cognitive impairment. It highlighted the necessity of taking both the two risk factors into consideration in cognitive assessment, prevention, and treatment of cognitive decline.

## Data Availability Statement

The raw data supporting the conclusions of this article will be made available by the authors, without undue reservation.

## Ethics Statement

The studies involving human participants were reviewed and approved by the Ethics Committee of Peking University People's Hospital. Written informed consent for participation was not required for this study in accordance with the national legislation and the institutional requirements.

## Author Contributions

YF collected, analyzed, interpreted the patient data, and was a major contributor in writing the manuscript. MS and XG made contributions to the acquisition of data. YH and CL helped in the statistical analysis of the data. RZ revised the manuscript and helped to interpret the data. JZ designed this study. All authors have read and approved the final version of this manuscript.

## Funding

This study was supported by the medical service and support capacity improvement project from the National Health Commission (Project title: The Relationship Between RBD and Cognition, Grant No. 2199000731) and the research project from the Beijing Natural Science Foundation (Grant No. 7192211).

## Conflict of Interest

The authors declare that the research was conducted in the absence of any commercial or financial relationships that could be construed as a potential conflict of interest.

## Publisher's Note

All claims expressed in this article are solely those of the authors and do not necessarily represent those of their affiliated organizations, or those of the publisher, the editors and the reviewers. Any product that may be evaluated in this article, or claim that may be made by its manufacturer, is not guaranteed or endorsed by the publisher.

## Abbreviations

MRI: magnetic resonance imaging
cSVD: cerebral small vessel disease
AD: Alzheimer's disease
VaD: vascular dementia
MMSE: Mini-Mental State Examination
MoCA: Montreal Cognitive Assessment
MTA: medial temporal atrophy
WMH: white matter hyperintensities
EPVS: enlarged perivascular space.
